# Dietary and sex-specific factors regulate hypothalamic neurogenesis in young adult mice

**DOI:** 10.3389/fnins.2014.00157

**Published:** 2014-06-13

**Authors:** Daniel A. Lee, Sooyeon Yoo, Thomas Pak, Juan Salvatierra, Esteban Velarde, Susan Aja, Seth Blackshaw

**Affiliations:** ^1^Solomon H. Snyder Department of Neuroscience, Johns Hopkins University School of MedicineBaltimore, MD, USA; ^2^Division of Biology and Biomedical Engineering, California Institute of TechnologyPasadena, CA, USA; ^3^Department of Radiation Oncology and Molecular Sciences, Johns Hopkins University School of MedicineBaltimore, MD, USA; ^4^Center for Metabolism and Obesity Research, Johns Hopkins University School of MedicineBaltimore, MD, USA; ^5^Institute for Cell Engineering, Johns Hopkins University School of MedicineBaltimore, MD, USA; ^6^Department of Ophthalmology, Johns Hopkins University School of MedicineBaltimore, MD, USA; ^7^Center for High-Throughput Biology, Johns Hopkins University School of MedicineBaltimore, MD, USA

**Keywords:** hypothalamus, neurogenesis, stem cells, median eminence, arcuate nucleus, dietary factors, sexual dimorphism, tanycytes

## Abstract

The hypothalamus is the central regulator of a broad range of homeostatic and instinctive physiological processes, such as the sleep-wake cycle, food intake, and sexually dimorphic behaviors. These behaviors can be modified by various environmental and physiological cues, although the molecular and cellular mechanisms that mediate these effects remain poorly understood. Recently, it has become clear that both the juvenile and adult hypothalamus exhibit ongoing neurogenesis, which serve to modify homeostatic neural circuitry. In this report, we share new findings on the contributions of sex-specific and dietary factors to regulating neurogenesis in the hypothalamic mediobasal hypothalamus, a recently identified neurogenic niche. We report that high fat diet (HFD) selectively activates neurogenesis in the median eminence (ME) of young adult female but not male mice, and that focal irradiation of the ME in HFD-fed mice reduces weight gain in females but not males. These results suggest that some physiological effects of high fat diet are mediated by the stimulation of ME neurogenesis in a sexually dimorphic manner. We discuss these results in the context of recent advances in understanding the cellular and molecular mechanisms that regulate neurogenesis in postnatal and adult hypothalamus.

## Introduction

Obesity and metabolic disorders are severe public health problems in developed countries. The pathophysiological effects of metabolic disease are at least partially mediated by hypothalamic inflammation (Thaler et al., 2012; Cai, 2013; Purkayastha and Cai, 2013), and by compensatory changes in hypothalamic neural circuitry resulting from obesity-induced neural injury. Supporting these observations, recent human anatomical scans have revealed hypothalamic neural injury in obese patients (Thaler et al., 2012). A fundamental understanding of the cellular responses to hypothalamic injury induced by dietary factors may provide new therapeutic targets for treating obesity and metabolic disorders (Lee and Blackshaw, 2012).

Newborn neurons in the postnatal and adult hypothalamus have been described in various vertebrate species (zebrafish Wang et al., 2012, hamster Mohr and Sisk, 2013, mouse Lee et al., 2012, and sheep Migaud et al., 2010), suggesting a degree of plasticity that is evolutionarily conserved, and likely extends to humans as well (Dahiya et al., 2011; Batailler et al., 2013). Both juvenile and adult mammalian hypothalamus exhibit ongoing neurogenesis that can be modulated by growth and differentiation factors (Pencea et al., 2001; Kokoeva et al., 2005; Xu et al., 2005; Perez-Martin et al., 2010; Robins et al., 2013a), diet (Lee et al., 2012; Li et al., 2012; McNay et al., 2012; Gouaze et al., 2013), and hormones (Ahmed et al., 2008). Although these studies generally agree that levels of constitutive neurogenesis are low (Lee and Blackshaw, 2012), they often report differing effects of extrinsic factors on cell proliferation and neurogenesis in the hypothalamus. Of note, these studies make opposing claims about levels of neurogenesis and proliferation in certain hypothalamic regions, and the cell(s) of origin for these adult-born neurons remain controversial (Lee and Blackshaw, 2014).

For instance, using a combination of *in vitro* cell culture and *in vivo* genetic lineage analysis, it has been claimed that a population of Sox2-positive (Li et al., 2012) and/or NG2-positive progenitors in the mediobasal hypothalamic parenchyma (Robins et al., 2013b) act as multipotent neural progenitors. Tanycytes of the hypothalamic ventricular zone have also been reported to act as neural progenitors (Xu et al., 2005; Lee et al., 2012; Li et al., 2012; Haan et al., 2013; Robins et al., 2013a), and it has been variously claimed that dorsally located alpha2 and ventral beta2 tanycytes of the median eminence show greatest levels of neurogenic potential (Lee et al., 2012; Haan et al., 2013; Robins et al., 2013a). The results of the studies employing cell lineage analysis to identify putative neural progenitor cell types in the postnatal hypothalamus are summarized in Table 1.

**Table 1:** **Summary of methodological details of recent studies that have used prospective lineage analysis in mice to identify the cell of origin of postnatally-generated hypothalamic neurons**.

**Cre line**	**Reporter**	**Sex**	**Proposed neural progenitor cell**	**Age of induction**	**Chase duration (days)**	**Tamoxifen delivery**	**Neuronal marker**	**References**
Nestin:CreER transgene	R26YFP	Both	Beta2 tanycytes	P4	30	Single 0.2 mg, i.p.	HuC/D	Lee et al., 2012
Glast:CreER transgene	R26LacZ	Both	Alpha2 tanycytes	P56–P84	42, 270	2 mg, 1x/days, 10 days	NeuN	Robins et al., 2013a
Sox2 promoter- Cre lentivirus	R26YFP	Male	Unknown parenchymal cell	P90	80	N/A	NeuN	Li et al., 2012
Fgf10:CreER knock-in	R26IacZ	N.S.	Beta tanycytes	P28, P57	39–83	100 mg/kg i.p, 1x/days, 7 days, then oral for 10 days	NeuN	Haan et al., 2013
NG2:CreER transgene	Ai9	Male	OPC	P56–P84	60d	1.2–1.5 mg, 2x/day, 5 days	NeuN, HuC/D	Robins et al., 2013b

*Abbreviations used: N.S., not specified*.

Other studies have reported that a range of extrinsic factors—such as dietary and hormonal signals, as well as growth and differentiation factors—can also modulate postnatal hypothalamic neurogenesis. High-fat diet (HFD) has been reported to constitutively inhibit neurogenesis in the mediobasal hypothalamic parenchyma (Li et al., 2012; McNay et al., 2012), while activating neurogenesis in the median eminence (Lee et al., 2012; Hourai and Miyata, 2013). It has also been reported that neurogenesis occurs in a sexually dimorphic pattern during puberty in hypothalamic regions, such as the preoptic area and anteroventral periventricular nucleus that control sexual behavior (Ahmed et al., 2008), although the source of these young adult-generated neurons was not investigated.

Although these results seem discrepant at first glance, a closer examination reveals that these observed effects may result from methodological differences among the studies, which are summarized in Table 2 (Migaud et al., 2010; Lee and Blackshaw, 2012). For instance, while multiple groups have reported that long-term administration of HFD inhibits cell proliferation and neurogenesis in hypothalamic parenchyma (Li et al., 2012; McNay et al., 2012; Gouaze et al., 2013), studies investigating acute responses to HFD have reported increased hypothalamic cell proliferation and neurogenesis (Thaler et al., 2012; Gouaze et al., 2013). Acute HFD administration has also been reported to rapidly induce hypothalamic inflammation, resulting in increased cytokine signaling (Thaler et al., 2012). The physiological response in acute vs. chronic HFD administration may serve different, but equally important, roles in maintaining metabolic homeostasis.

**Table 2:** **Summary of methodological differences among studies reporting differential regulation of hypothalamic neurogenesis by extrinsic factors**.

**Species**	**Sex**	**Region**	**BrdU dosage**	**Age injected**	**Chase time (days)**	**Treatment**	**Treatment onset**	**Treatment duration**	**Neuronal marker**	**Effect on neurogenesis**	**References**
Mouse	Male	ArcN	0.5 μgl/h, 7 days, i.c.v.	P56	3	HFD	P60	3 days	NeuN, Pomc	Increase	Gouaze et al., 2013
Mouse	Male	ArcN	0.5 μgl/h, 7 days, i.c.v.	P112	28	HFD	8 weeks	56–140 days	HuC/D	Decrease	McNay et al., 2012
Mouse	Male	ArcN	50 mg/kg 7 days, i.p.	P45	30	HFD	P42	33 days	HuC/D	Decrease	Current study
Mouse	Female	ArcN	50 mg/kg, 2x/day, 9 days, i.p.	P45	30	HFD	P42	33 days	HuC/D	Decrease	Current study
Mouse	Male	ArcN	10 μg/day, 7 days, i.c.v.	P210	30	HFD	P90	120 days	NeuN, Pomc	Decrease	Li et al., 2012
Mouse	Male	Widespread	12 μg/day, 7 days, i.c.v.	P56	30	CNTF	P56	60–90 days	HuC/D, NeuN	Increase	Kokoeva et al., 2005, 2007
Mouse	Male	ME	50 mg/kg, 2x/day, 9 days, i.p.	p45	30	HFD	P42	33 days	HuC/D	No change	Current study
Mouse	Female	ME	50 mg/kg, 2x/day, 9 days, i.p.	p45	30	HFD	P42	33 days	HuC/D	Increase	Lee et al., 2012; current study
Mouse	Female	ME	50 mg/kg, 2x/day, 9 days, i.p.	p45	30	HFD/CR	P42	33 days	HuC/D	Decrease vs. HFD	Current study
Rat	Female	AVPV/ MPOA	300 mg/kg. 3 days, i.p.	P30	20	Gndx	P20	n/a	NeuN	Decrease	Ahmed et al., 2008
Rat	Male	AVPV/ MPOA	300 mg/kg. 3 days, i.p.	P30	20	Gndx	P20	n/a	NeuN	No change	Ahmed et al., 2008
Rat	Both	Periventricular	q2h for 48 h (50 mg/kg), i.p.	P56	28	bFGF	8 weeks	Single injection	HuC/D	Increase	Xu et al., 2005
Rat	N.S.	Widespread	12 mg/day, 7d, i.c.v	Adult	28	BDNF (i.c.v)	Adult	Adult+12 days	TuJ1, MAP2	Increase	Pencea et al., 2001

*Abbreviations used: AVPV, anterioventral periventricular nucleus; Gndx, gonadectomy, N.S., not specified; POA, preoptic area*.

Hypothalamic progenitor cell populations may likewise respond differentially, and in some cases with opposite reactions, to dietary signals such as HFD. The median eminence (ME), for instance, lies outside the blood-brain barrier; it is thus exposed to higher effective concentrations of circulating dietary and hormonal cues than the hypothalamic parenchyma (Fry et al., 2007; Langlet et al., 2013b). In contrast, all tanycyte subtypes directly contact the CSF, and can potentially respond to intracerebral ventricular signals (Bennett et al., 2009; Bolborea and Dale, 2013).

The age of the mice used for these studies has ranged from early postnatal (Lee et al., 2012), to young adult (Ahmed et al., 2008; Lee et al., 2012), to 3–12 months of age(Kokoeva et al., 2005, 2007; Lee et al., 2012; McNay et al., 2012). Finally, studies of postnatal and adult neurogenesis in the ventrobasal hypothalamus have used either only male (Kokoeva et al., 2005, 2007; Li et al., 2012; McNay et al., 2012) or only female (Lee et al., 2012) mice. Neurogenesis in other hypothalamic regions is sexually dimorphic (Ahmed et al., 2008), making this but one additional methodological difference that could contribute to differences in the levels, location and dietary regulation of hypothalamic neurogenesis reported in these studies.

To clarify the extent to which sex-dependent factors might regulate neurogenesis in different hypothalamic regions, we investigated levels of hypothalamic neurogenesis in both the arcuate nucleus (ArcN) and ME in male and female young adult mice fed normal chow and HFD. We also investigated the effects of low-protein diet (LPD) and caloric restriction (CR) in these same areas in female mice. These dietary treatments led to significant and region-specific differences in neurogenesis. Most notably, HFD treatment inhibited ArcN neurogenesis in both sexes, while selectively stimulating ME neurogenesis in female mice. In mice fed HFD, we found that inhibiting ME neurogenesis by computer tomography-guided focal irradiation attenuated weight gain in females but not males. These findings advance our understanding of physiological factors that regulate adult hypothalamic neurogenesis, and reconcile a number of seemingly discrepant recent studies on this topic.

## Results

### Dietary signals differentially regulate neurogenesis and cell proliferation in the hypothalamic median eminence and arcuate nucleus

**Figure 1:**
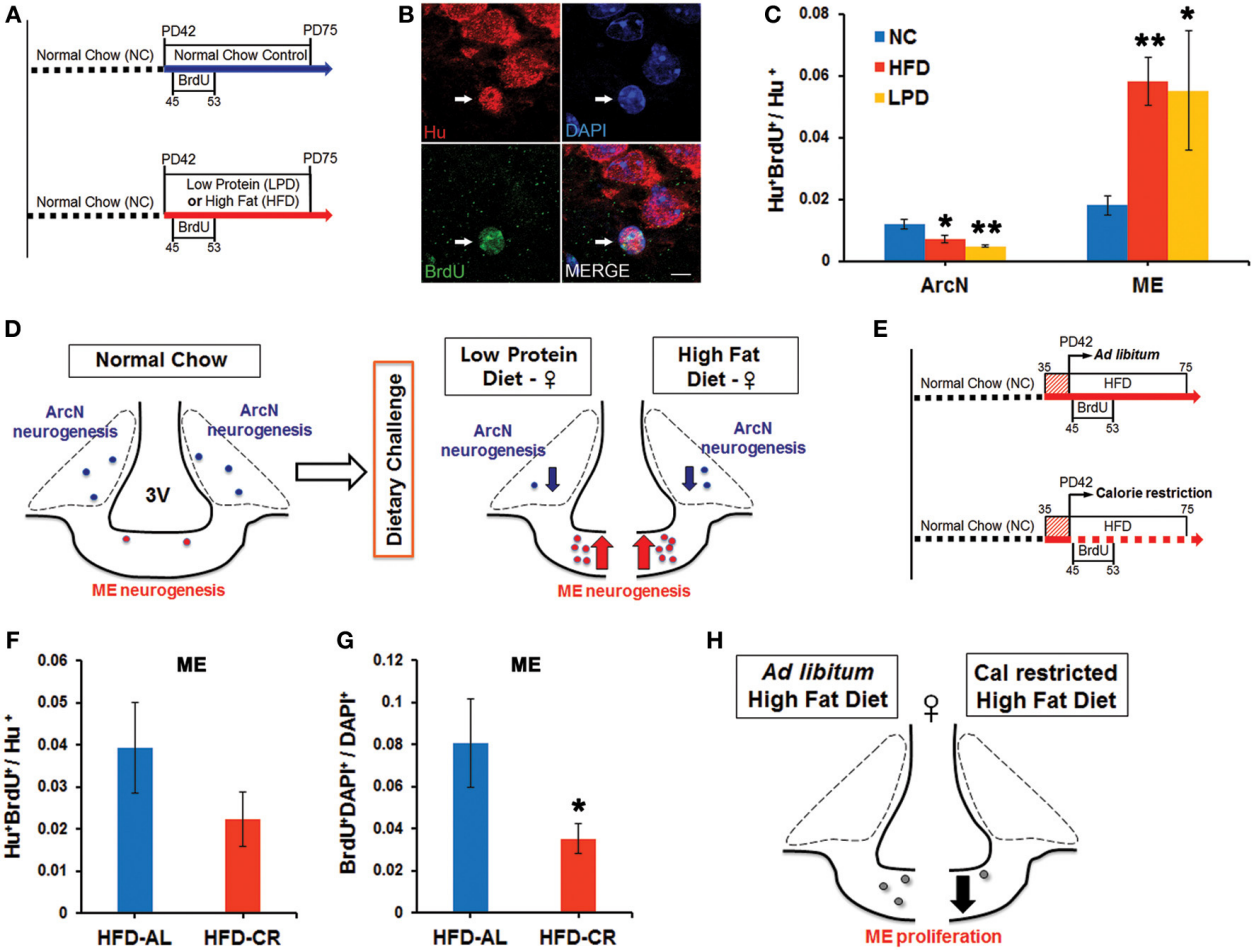
**Dynamic alteration of hypothalamic neurogenesis in response to different dietary conditions**. **(A)** Experimental design schematic. Female mice were either continuously fed on normal chow (NC), or switched to the low protein diet (LPD) or high fat diet (HFD) at postnatal day (PD) 42. After three days, BrdU was injected intraperitoneally twice per day for 9 days. Mice were sacrificed 1 month after the onset of BrdU administration. **(B)** Representative high magnification image of BrdU and Hu double-positive P75 adult-born neurons (white arrows) located in median eminence (ME) of female mice fed HFD. **(C)** Quantitative comparison of diet-dependent neurogenesis (Hu^+^BrdU^+^/Hu^+^ neurons) in the arcuate nucleus (ArcN) and median eminence (ME). **(D)** Schematic summarizing opposite effects of dietary change on neurogenesis between ArcN and ME. **(E)** Scheme of experimental design for calorie restriction. After 1 week initial adjustment to HFD (red striped square), female mice were fed either *ad libitum* (AL) HFD, or were calorie restricted (CR) on the HFD to 70% of the *ad libitum*-fed mice, from PD42 onward. After three days, BrdU was injected intraperitoneally twice per day for 9 days. Mice were sacrificed 1 month after BrdU administration onset. **(F,G)** Quantitative comparison of ME neurogenesis (Hu^+^BrdU^+^/Hu^+^ neurons) and proliferation (BrdU^+^DAPI^+^/DAPI^+^ cells) in calorically restricted HFD-fed (HFD-CR) mice or *ad libitum* HFD-fed (HFD-AL) mice. **(H)**. Schematic summarizing significant reduction of cell genesis in the ME of mice fed on HFD-CR. ^*^p < 0.05, ^**^p < 0.003. Scale bar: 5 μm.

Our group previously demonstrated that feeding HFD to young adult female mice led to significantly increased neurogenesis in the median eminence (ME) (Lee et al., 2012). We set out to test whether additional dietary conditions could also alter neurogenesis in the ME, and whether these led to comparable changes in neurogenesis in the arcuate nucleus (ArcN), which lies inside the blood-brain barrier within the hypothalamus proper. Young adult female mice were continued on normal chow (NC), or switched to HFD or LPD beginning at postnatal day (P) 42. Cell proliferation was tracked using twice-daily intraperitoneal (i.p.) injections of BrdU from P45-53 (Figure 1A). Mice were euthanized at P75, and brains were immunostained for BrdU and the pan-neuronal marker HuC/D. The fraction of Hu^+^ cells that were also BrdU^+^ was quantified to assess levels of neurogenesis.

Baseline levels of hypothalamic neurogenesis [(Hu^+^BrdU^+^)/Hu^+^ neurons: mean ± s.e.m] in mice fed NC were low and did not differ significantly between the two regions [ArcN (0.012 ± 0.002, *n* = 5) vs. ME (0.015 ± 0.004, *n* = 7), *p* = 0.47] (Figure 1C). Both the HFD-fed mice (0.0072 ± 0.0012, *n* = 5, *p* = 0.044) and LPD-fed mice (0.0048 ± 0.0004, *n* = 5, *p* = 0.0022) showed a substantial reduction in the fraction of Hu^+^BrdU^+^ ArcN neurons compared to NC-fed mice. In contrast, both HFD-fed mice (0.058 ± 0.008, *n* = 9, *p* = 0.0005) and LPD-fed mice (0.055 ± 0.019, *n* = 4, *p* = 0.025) showed a significant increase in the fraction of Hu^+^BrdU^+^ ME neurons (Figure 1C). The differences in neurogenesis levels between the ArcN and ME following both HFD [ArcN (0.0072 ± 0.001, *n* = 5) vs. ME (0.058 ± 0.008, *n* = 9), *p* = 0.0005] and LPD [ArcN (0.0048 ± 0.0004, *n* = 5) vs. ME (0.055 ± 0.019, *n* = 4), *p* = 0.021] were significant, and imply that neural progenitor populations in these two regions respond differentially to these dietary cues (Figures 1C,D). The HFD-induced inhibition of ArcN neurogenesis is similar to observations of adult male mice by other groups (Li et al., 2012; McNay et al., 2012).

Because previous studies reported that caloric restriction could reverse the effects of HFD on ArcN neurogenesis (McNay et al., 2012), we next tested whether caloric restriction could likewise modulate HFD-induced ME neurogenesis. For these studies, female mice were housed individually and allowed either *ad libitum* or restricted access to HFD starting at P42. Restricted HFD access was at 70% of the caloric intake of animals fed *ad libitum* (Figure 1E). BrdU labeling and immunohistochemistry were conducted as described above. Caloric-restricted HFD-fed mice trended toward a decrease in neurogenesis levels in the ME [HFD ad lib (0.039 ± 0.011, *n* = 4) vs. HFD-CR (0.022 ± 0.006, *n* = 6) *p* = 0.19], but this effect did not reach significance (Figure 1F). However, we observed that overall BrdU incorporation in ME cells was significantly reduced [HFD ad lib (0.08 ± 0.02, *n* = 4) vs. HFD-CR (0.035 ± 0.007, *n* = 6), *p* = 0.043] (Figure 1G). These results suggest that caloric restriction inhibits cell genesis in the median eminence (Figure 1H).

### Sex-specific differences in diet-induced hypothalamic neurogenesis

**Figure 2:**
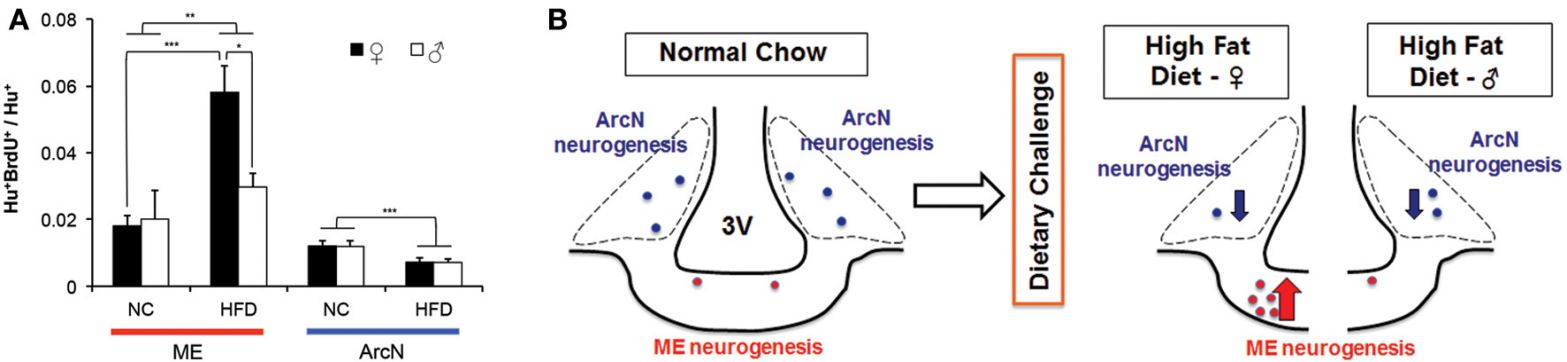
**Sex differences in neurogenic response to high fat diet**. Mice were maintained on normal chow, or challenged with a HFD *ad libitum* at 6 weeks old. At 6.5 weeks old, mice received BrdU intraperitoneally from P45-53, and were euthanized at 10.5 weeks old. **(A)** Adult born hypothalamic neurons (Hu^+^BrdU^+^/Hu^+^) were quantified in the median eminence (ME) and the arcuate nucleus (ArcN) under NC or HFD conditions. Two-Way ANOVA demonstrated a significant main effect of diet on ArcN neurogenesis [*F*_(1, 14)_ = 11.911, *p* = 0.004], as well as on ME neurogenesis, *F*_(1, 18)_ = 8.696, *p* = 0.009. However, no main effect was observed with gender alone on ME neurogenesis, *F*_(1, 18)_ = 2.444, *p* = 0.135. Analyses further revealed there was a significant interaction between diet and gender on ME neurogenesis, however, with *F*_(1, 18)_ = 4.409, *p* = 0.050. *Post-hoc* analyses using Holm-Sidak contrast indicated that within females, there was a significant effect on the levels of ME neurogenesis in high fat diet fed (0.058 ± 0.006) vs. normal chow fed (0.016 ± 0.007, *p* < 0.001), whose effect was not observed within the male cohort (*p* = 0.624). Likewise, *post-hoc* analyses indicated that within high fat treated mice, the mean levels of ME neurogenesis in female mice (0.058 ± 0.006) were significantly higher than that observed in male mice (0.027 ± 0.010) (Difference of means = 0.031, *t* = 2.636, *p* = 0.017). The mean level of ME neurogenesis within normal chow fed mice, however, do not differ between male (0.020 ± 0.010) and female (0.016 ± 0.007, *p* = 0.714). (^*^p < 0.025; ^**^p < 0.01; ^***^p < 0.005). **(B)** Schematic summarizing sexual dimorphism of dietary challenge on ME neurogenesis.

Since these aforementioned studies were all performed in young adult females, we next tested whether the levels of baseline and HFD-induced hypothalamic neurogenesis were different between the sexes. To directly compare results between males and females, we used diet and BrdU labeling conditions identical to those detailed in Figure 1A to measure levels of neurogenesis in male mice fed either NC or HFD. Two-Way ANOVA analysis, considering both diet and gender, showed that there was a significant main effect of diet on ArcN neurogenesis, *F*_(1, 14)_ = 11.911, *p* = 0.004 (Figure 2A). *Post-hoc* analyses using the Holm-Sidak method indicated that mice fed HFD showed significantly reduced levels of ArcN neurogenesis relative to mice fed NC in both sexes [Male: NC ArcN (0.012 ± 0.0017, *n* = 3) vs. HFD ArcN (0.0070 ± 0.0013, *n* = 5), *p* = 0.038; Female: NC ArcN (0.012 ± 0.0013, *n* = 5) vs. HFD ArcN (0.0072 ± 0.0013, *n* = 5), *p* = 0.020]. However, there was no significant interaction between gender and diet in levels of neurogenesis in the ArcN [Two-Way ANOVA, *F*_(1, 14)_ = 0.00046, *p* = 0.98].

Two-Way ANOVA analysis of levels of neurogenesis in the ME found a significant main effect of diet on ME neurogenesis, *F*_(1, 18)_ = 8.696, *p* = 0.009 (Figure 2A). *Post-hoc* analysis indicated that, in females, the mean levels of neurogenesis in mice fed on HFD (0.058 ± 0.006) were significantly higher than that observed in mice fed NC (0.016 ± 0.007) (*p* < 0.001), whose effect was not observed within male cohort (NC [0.02 ± 0.01] vs. HFD [0.027 ± 0.01], *p* = 0.624). No main effect was observed with gender alone on ME neurogenesis, *F*_(1, 18)_ = 2.444, *p* = 0.135. However, *post-hoc* analysis revealed that in the HFD-fed cohort, females showed significantly higher levels of ME neurogenesis than male [female (0.058 ± 0.006) vs. male (0.027 ± 0.01), *p* = 0.017], while there was not a difference in levels of ME neurogenesis between male and female in the NC-fed cohort [male (0.020 ± 0.010) vs. female (0.016 ± 0.007), *p* = 0.714]. Further analysis revealed there was a significant interaction between diet and gender on regulation of ME neurogenesis, *F*_(1, 18)_ = 4.409, *p* = 0.050, indicating that HFD-induced activation of ME neurogenesis is sexually dimorphic.

In addition, Two-Way ANOVA analysis comparing the effects of sex and hypothalamic region on the levels of neurogenesis in each diet condition revealed several significant effects. Levels of neurogenesis in both ArcN and ME were low in the NC cohort, and did not show sex-dependent differences [Two-Way ANOVA, *F*_(1, 14)_ = 0.280, *p* = 0.61]. Within the HFD-fed cohort, there was significant main effects of sex [*F*_(1, 18)_ = 4.463, *p* = 0.049], hypothalamic region [*F*_(1, 18)_ = 23.339, *p* < 0.001], and a marginally significant sex × hypothalamic region interaction [*F*_(1, 18)_ = 4.359, *p* = 0.051]. *Post-hoc* testing showed that significantly different levels of neurogenesis between ME and ArcN were observed in females [ME (0.058 ± 0.0053) vs. ArcN (0.0072 ± 0.0072), *p* < 0.001], but not in males [ME (0.027 ± 0.0092) vs. ArcN (0.0070 ± 0.0072), *p* = 0.101]. These results confirmed that HFD-dependent modulation of neurogenesis is sexually dimorphic in the ME but not the ArcN (Figures 2A,B).

### Blocking neurogenesis in the median eminence attenuates HFD-induced weight gain in young adult female, but not male, mice

What is the physiological role of these adult-generated hypothalamic neurons? Previous studies (Kokoeva et al., 2005; Gouaze et al., 2013) have attempted to address the functional role of these newborn hypothalamic neurons through chemical suppression of hypothalamic neurogenesis with molecular reagents, such as arabinofuranosyl cytidine (also known as AraC), a chemotherapy compound that broadly inhibits DNA synthesis within the brain or body depending on the administration route. This lack of hypothalamic specificity, however, makes it difficult to attribute corresponding physiological alterations with the function of hypothalamic neurogenesis. In order to selectively suppress proliferation/neurogenesis of neural progenitors residing in the hypothalamic median eminence, we developed a computer tomography-guided focal irradiation methodology. This focal irradiation approach affords a high degree of spatial specificity, allowing us to selectively target ME neural progenitors in contrast to chemical suppression approaches. While the biological basis for the suppression of proliferation/neurogenesis in neural progenitors after irradiation still remains unclear (Monje et al., 2002), recent studies of the classic neurogenic region in the hippocampus have demonstrated that highly focal irradiation with a dose of 10 Gy can suppress neurogenesis for at least 4 weeks after irradiation (Ford et al., 2011).

**Figure 3:**
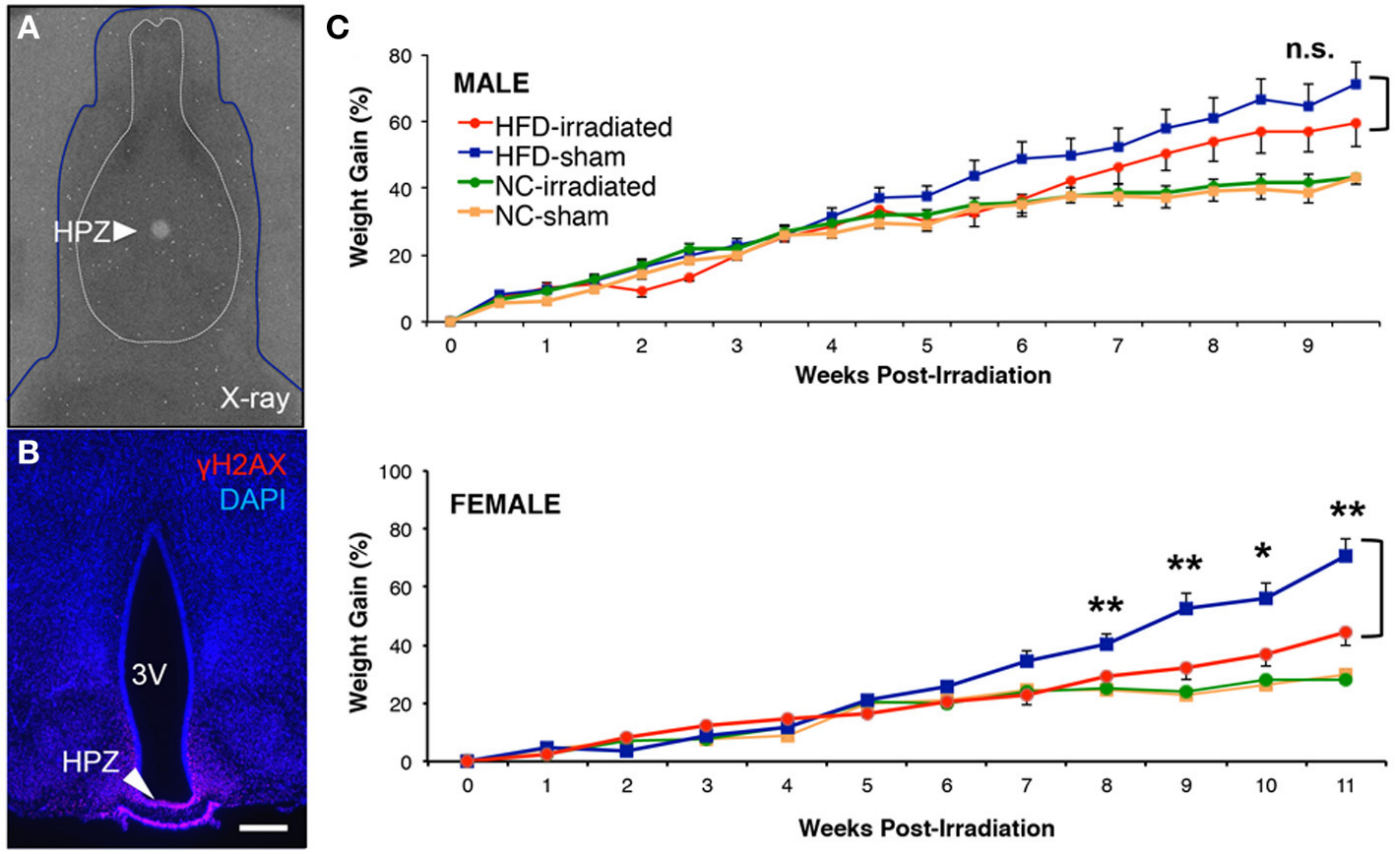
**Sex differences in diet-induced weight gain after focal inhibition of median eminence neurogenesis**. Mice received either a high fat diet *ad libitum* at 5.5 weeks old, or were kept on normal chow control diet. At 6 weeks old, sham treatment or 10 Gy of computer tomography-guided focal radiation was targeted to the hypothalamic median eminence of young adult mice as previously detailed in the online JoVE protocol (Lee et al., 2013). **(A)** Superimposition of dosimetry-film acquired with 1-mm radiation beam in phantom with an X-ray of a real mouse subject (blue line). White circle (arrow) indicates 10-Gy dose of radiation that has been focally targeted to HPZ. **(B)** Confirmation of radiation targeting accuracy by γH2AX immunostaining, an indicator of DNA double strand breaks and radiation localization. **(C)** Weight gain was normalized to weight at the time of either sham or focal irradiation treatment in NC- and HFD-fed mice of both sexes. There was a significant interaction between diet and treatment only in female mice (a Two-Way ANOVA, n.s., not significant, *p* = 0.363; ^*^p < 0.05; ^**^p < 0.01).

We demonstrate that computer-tomography guided focal irradiation can selectively inhibit cell proliferation in the ME, while sparing proliferation in the ArcN (Lee et al., 2012), and we provide an extensively detailed video and written protocol in the *Journal of Visualized Experiments* (Lee et al., 2013). Our radiological approach reduces ME neurogenesis by ~85% (Lee et al., 2012), in line with previous approaches that used focal irradiation in other mammalian neurogenic niches (Ford et al., 2011). Using this focal irradiation approach, we tested whether selective radiological inhibition of ME neurogenesis (Figure 3A) in both males and female mice fed HFD led to sex-specific differences in regulation of body weight. Dietary change to HFD was initiated at 5.5 weeks of age, and irradiation (or sham) was performed with 6 week old mice. The specificity of the focal irradiation was demonstrated using γH2AX immunostaining, a marker of double-strand DNA breaks (Figure 3B; Lee et al., 2012, 2013). Longitudinal body weight measurements were then taken for male and female mice that underwent either focal irradiation or sham treatment, and were fed either NC or HFD (Figure 3C). Weight changes were normalized to the starting weight of each mouse at the time of sham or irradiation treatment.

We observed no long-term differences in body weight gained between sham and irradiated animals fed normal chow in either our male (sham treatment: *n* = 8; irradiation treatment: *n* = 12) or female (sham treatment: *n* = 12; irradiation treatment: *n* = 12) cohorts (Figure 3C). In contrast, HFD-fed female mice showed a significant reduction in weight gain following irradiation relative to sham controls, as previously reported (Lee et al., 2012). At 9 weeks post-treatment, irradiated female mice receiving HFD (*n* = 10) had 32 ± 4% increase in weight gain relative to sham controls (*n* = 9), which showed a 52 ± 6% increase in weight gain (student *t*-test: *p* = 0.028 Figure 3C). In addition, Two-Way ANOVA analysis revealed that a significant interaction between diet and treatment was detected after 8 weeks post-treatment (*p* = 0.009) and maintained up to the end of weight taking at week 11 in female mice (week 9, *p* = 0.009; week 10, *p* = 0.037; week 11, *p* = 0.004). In sharp contrast, no significant differences in weight gain were observed in irradiated HFD-fed males relative to sham controls throughout the 10 weeks of weight taking [at week 10, irradiated (59 ± 7% increase, *n* = 11) vs. sham (71 ± 7% increase, *n* = 12), student *t*-test: *p* = 0.27, Figure 3C] with no significant interaction between diet and treatment (Two-Way ANOVA at week 10, *p* = 0.36). These data confirm previous reports that adult-born neurons generated in the female ME in response to HFD act to promote energy storage (Lee et al., 2012, 2013).

## Discussion

**Figure 4:**
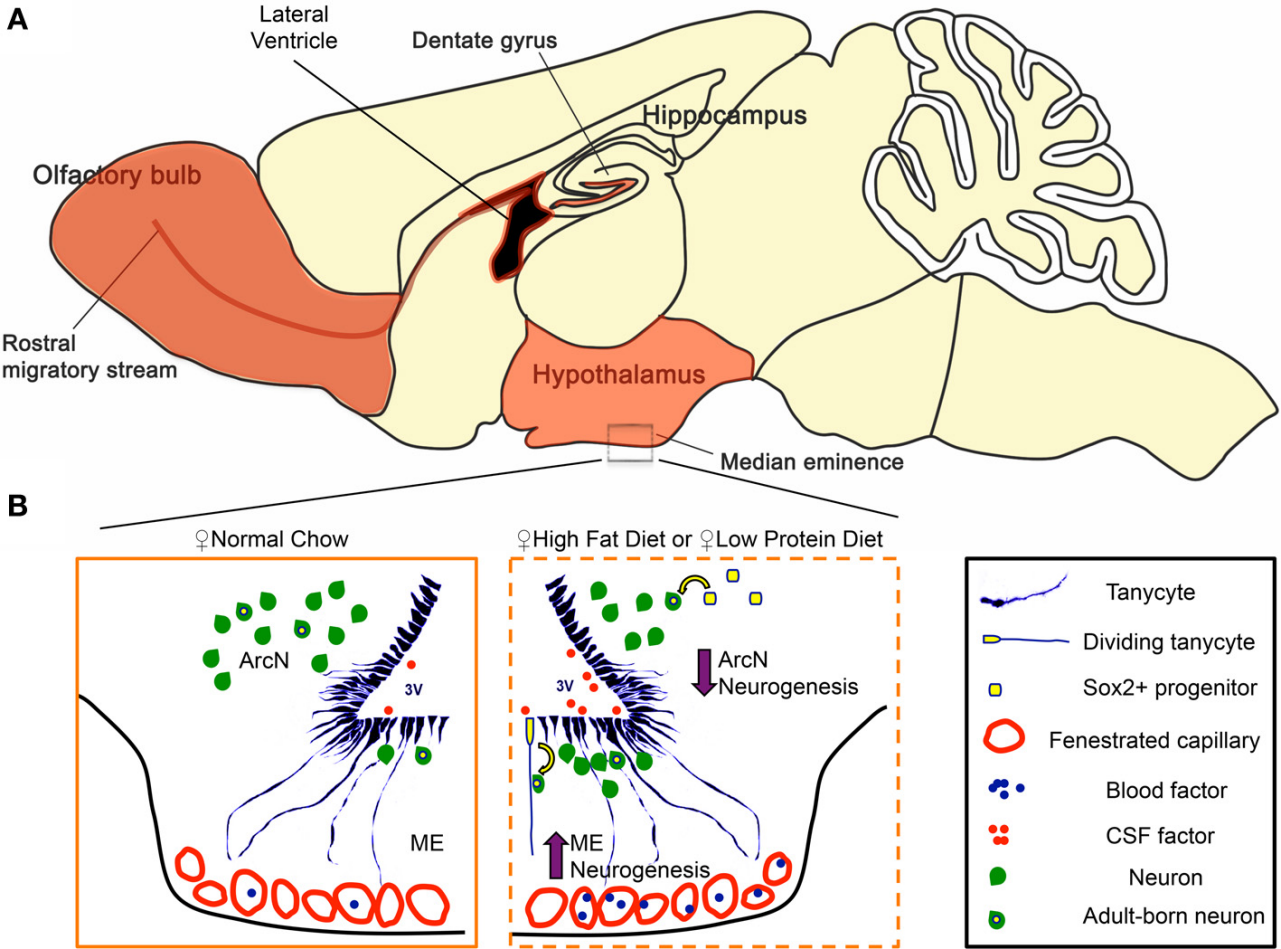
**Regulation of hypothalamic neurogenesis by dietary factors. (A)** In mammals, constitutive adult neurogenesis is primarily confined to three brain regions (highlighted in orange). The hippocampal dentate gyrus and the subventricular zone of the lateral ventricles are canonical neurogenic niches. Additionally, recent observations demonstrate that the ventrobasal hypothalamus serves as a neurogenic niche, engaging in low but constitutive levels of neurogenesis in adults. **(B)** Hypothalamic tanycytes and parenchymal Sox2^+^ cells represent potential hypothalamic neural progenitor populations that give rise to adult-born neurons. In the hypothalamic median eminence (ME), tanycytes are a convincing neurogenic source for the arcuate nucleus (ArcN) and ME. In females, both high-fat (HFD) and low protein diet (LPD) increase ME neurogenesis in the ME, while concurrently decreasing ArcN neurogenesis in the ArcN. Several lines of evidence suggest that this dynamic change in neurogenesis is mediated between a tanycytic neural progenitor pool, and factors present in the cerebrospinal fluid (CSF) and/or those circulating through the fenestrated capillaries of the ME.

Several recent studies have reported that neurogenesis occurs in the adult hypothalamus (Migaud et al., 2010; Lee and Blackshaw, 2012, 2014), a central regulator of metabolism and energy balance (Figure 4A). We investigated how changes in diet can modulate hypothalamic neurogenesis by presenting young adult mice with contrasting diets. We observed that HFD, LPD, and caloric-restricted HFD all differentially modulate cell genesis in the hypothalamic median eminence and arcuate nucleus, hypothalamic regions that regulate energy balance (summarized in Figure 4B). In both sexes, we observed a decrease in ArcN neurogenesis in response to both HFD and LPD (Figures 1C,D). In contrast, HFD selectively enhances neurogenesis in the female ME, suggesting the existence of sex-specific modulation of neurogenesis limited to the ME in the mediobasal hypothalamus. The functional relevance of this female-specific change in the ME for energy balance was confirmed by diminished diet-induced obesity only in irradiated female mice.

The functional properties of adult-born neurons most likely differ in response to different dietary challenges, and this may be dependent on neuronal subtypes that are differentially generated in response to sex-specific and diet-dependent signals. In order to better classify the neuronal subtypes of the ME, we performed an *in silico* analysis of mediobasal hypothalamic neuronal subtype markers from our *in situ* screen (Shimogori et al., 2010), and the Allen Brain Atlas (Lein et al., 2007). Using three-dimensional correlative and cluster analyses features of the Anatomic Gene Expression Atlas interface (http://mouse.brain-map.org), we identified multiple genes that are candidates for selective labeling of neurons in the ArcN, as well as the ME, as determined by their expression in a subset of cells located immediately ventral to β 2 tanycytes of the ventricular zone of the ME. These candidate marker genes include neuropeptides known to modulate feeding and appetite (Supplementary Figures 1D–G), molecules mediating signaling by diet-regulated hormones (Irs4 and Leptin receptor, Supplementary Figures 1J,K), as well as secreted serine protease inhibitors (Serpina3c, Serpina3k), whose roles in inflammation are beginning to be elucidated. These observations suggest that the differential region-specific changes in neurogenesis may lead to differential generation of orexinergic (Supplementary Figures 1D,F) and anorexinergic neurons (Supplementary Figures 1E,G) in response to dietary cues, and may serve as a mechanism that allows adaptation to long-term changes in energy balance homeostasis.

These region-specific changes in adult hypothalamic neurogenesis most likely are mediated by differing exposures to secreted peptide, growth factors, and neurotrophic factors that signify feeding status and long-term energy availability. The median eminence, by virtue of its access to the third ventricle and status as a circumventricular organ, is exposed to a variety of these secreted factors via the cerebrospinal fluid and the blood. By comparison, the arcuate nucleus, a structure protected by the blood-brain-barrier (Mullier et al., 2010), has less access to circulating satiety signals and hormones. The permeability of hypothalamic blood brain barrier to blood-borne factors is differentially regulated in fed and fasting conditions through a VEGF-dependent mechanism (Langlet et al., 2013a). Continuous integration of these peripheral signals by neurons belonging to both the arcuate nucleus and the median eminence of the hypothalamus is critical for central regulation of energy balance and neuroendocrine function (Schaeffer et al., 2013). Our data suggest that adult-generated neurons in both hypothalamic regions may show differing sensitivities to dietary and hormonal signals that help maintain energy homeostasis.

Several secreted factors that signal feeding status and long-term energy availability regulate adult neurogenesis in various neurogenic niches, including the hypothalamus (reviewed in Table 2) (Sousa-Ferreira et al., 2014). For instance, compared to normal chow fed controls, HFD-fed mice exhibited substantially higher ciliary neurotrophic factor (CNTF) mRNA in tanycytes and multi-ciliated ependymal cells, while calorically-restricted mice showed substantially lower expression levels; this coincided with similar changes in CNTF receptor (CNTFR) mRNA (Severi et al., 2013). Taken together with previous findings that CNTF delivered by i.c.v. cannulation stimulates adult hypothalamic neurogenesis (Kokoeva et al., 2005), this suggests that dietary signals may regulate hypothalamic neurogenesis in the ME through altered CNTF signaling. This is supported by observations that β 2 tanycytes of the adult hypothalamic proliferative zone (HPZ) are enriched with CNTFR, as compared to α1,2 tanycytes (Kokoeva et al., 2005). In that study, mice receiving intracerebroventricular infusion of CNTF demonstrated hyperplasia within the HPZ of the ME, as indicated by particularly high levels of BrdU incorporation (Kokoeva et al., 2005). The development of inducible Cre mouse lines specific for hypothalamic neural progenitors will help identify additional signaling pathways that are critical for the regulation of hypothalamic neurogenesis (Robins et al., 2013a; Pak et al., 2014).

In female mice fed a normal chow diet, we observed relatively low levels of hypothalamic neurogenesis in the median eminence. Interestingly, upon presentation of a dietary challenge such as high-fat diet, median eminence neurogenesis was substantially increased in females. What is the physiological role of adult born hypothalamic neurons? Converging lines of evidence suggest that the function of these newly generated neurons is the regulation of metabolism, energy balance, and weight (Bolborea and Dale, 2013; Lee and Blackshaw, 2014). In contrast to previous studies using AraC to inhibit cell proliferation in the hypothalamus (Kokoeva et al., 2005; Gouaze et al., 2013), which showed that inhibition of neurogenesis by AraC infusion may increase weight gain or prevent weight loss, we show that using computer-tomography guided focal irradiation, a highly selective and specific targeting method, we are able to inhibit neurogenesis in the median eminence (Lee et al., 2012, 2013). As a consequence to this radiological treatment, we observe alterations in weight gain in a subset of irradiated mice compared to sham controls, with reduced HFD-induced weight gain in females, but not in males (Figure 3C). These intriguing results suggest that weight gain in females can be attributed in part to additional adult-generated neurons in the ME, and that the neural circuitry regulating body weight differs in some respects between females and males. These findings are consistent with previous studies demonstrating that sex hormones can regulate hypothalamic neurogenesis in a region-dependent manner (Ahmed et al., 2008; Cheng, 2013). Taken together with our results, this body of work highlights the importance of examining both the regional differences in hypothalamic neurogenesis and the sex-specific differences. Such differences likely at least partially account for differences in the levels and diet-dependence of adult hypothalamic neurogenesis observed by different groups (Table 2).

Factors that mediate these sex-dependent differences in hypothalamic neurogenesis have not yet been identified, but could involve numerous levels of regulation, such as hormone-dependent plasticity (de Seranno et al., 2010), differences in blood-barrier access between the sexes (Hoxha et al., 2013), and hormone-specific induction of feeding behavior (Sieck et al., 1978). Lastly, it is possible that it is the survival of newborn neurons, rather than (or in addition to) their proliferation, which is sexually dimorphic, as has been previously demonstrated for prenatally generated hypothalamic neurons (Tobet and Hanna, 1997; Park et al., 1998; Forger et al., 2004; Waters and Simerly, 2009).

In addition to being a means of regulating energy homeostasis in response to long-term changes in diet, adult hypothalamic neurogenesis may be triggered in response to environmental injury. The hypothalamic median eminence, in contrast to other hypothalamic regions, lies outside of the blood-brain barrier, and is thus directly exposed to circulating toxins and pathogens, as well as nutrients that can lead to cellular injury when in oversupply. Hypothalamic neural injury and inflammation are seen in obese animals and humans (Li et al., 2012; Thaler et al., 2012, 2013). The increased neurogenesis in adult female ME may serve to replace damaged neurons in this region. Indeed, at least one study has reported that neurons important for energy balance regulation can be regenerated in adult hypothalamus in response to neurodegenerative-like injury (Pierce and Xu, 2010). Further studies to determine the role of environmental and physiological factors in regulating adult hypothalamic neurogenesis may yet reveal new mechanistic approaches toward the treatment of obesity and metabolic disorders.

## Materials and methods

### Animals

Five weeks old female or male C57BL/6 mice were obtained from Charles River and housed in a 14/10-h light-dark cycle with free access to normal chow (Teklad F6 Rodent Diet 8664:: Protein (kcal): 31%, Carbohydrate (kcal): 50%, Fat (kcal): 19%, Harlan Teklad, Madison, WI) and water. Where indicated, animals were provided with a high-fat diet (HFD) (Catalog #: D12492i:: Protein (kcal): 20%, Carbohydrate (kcal): 20%, Fat (kcal): 60%, Research Diets, New Brunswick, NJ) or low protein diet (Catalog#: D11112203:: Protein (kcal): 8%, Carbohydrate (kcal): 76%, Fat (kcal): 16%, Research Diets, New Brunswick, NJ). All mice used in these studies were maintained and euthanized according to protocols approved by the Institutional Animal Care and Use Committee at the Johns Hopkins School of Medicine.

### Caloric restriction

Five weeks old female C57BL/6 mice were obtained from Charles River and put on a high-fat diet (HFD: 60% kcals from fat, Research Diets, #D12492). At six weeks old, mice were separated into two groups: (control group) HFD *ad libitum* and HFD caloric restriction (CR). CR is at 70% of the HFD control group's average food intake. This was calculated by providing the amount of the control group's average food intake, plus an amount equal to the standard error of that group's intake, to ensure that mice would have enough food both to eat, and to spill, and maintain 70% of the HFD control group's average food intake. Food intakes were measured twice per week and used to calculate the CR levels to be used for 0.5 weeks until the next food intake assessment.

### Reagents

#### Bromodeoxyuridine (BrdU)

Where indicated, young adult mice received bromodeoxyuridine (BrdU; Sigma) administrated in the morning and evening by intraperitoneal injection at 50 mg/kg of body weight from P45 to P53.

#### Tissue processing and antibodies used

Adult mice were sacrificed, perfused with 4% PFA/PBS, and cryoprotected as previously described (Lee et al., 2012). Serial sections (40 μm thick) were collected and stored at −20°C. Free-floating sections were immunostained using the following primary antibodies and working concentrations: mouse monoclonal anti-phospho-H2AX, Ser139, clone JBW301 (1:700, Millipore), rat monoclonal anti-BrdU (1:200, Accurate, Westbury, NY), mouse monoclonal anti-Hu (5 μg/ml, Molecular Probes, Carlsbad, CA). Double staining was visualized with Alexa Fluor 555-, and Alexa Fluor 488 (1:500, Molecular Probe, Carlsbad, CA). 4′,6-diamidino-2-phenylindole (DAPI) was used as a nuclear counterstain.

### Immunohistochemistry

γH2AX immunostaining was performed as previously described (23). For BrdU immunostaining, sections were first incubated in 2N HCl at 37°C for 30 min, and rinsed in 0.1 M boric acid (pH 8.5) at room temperature for 10 min. Sections were then rinsed in PBST, blocked for 5 min in SuperBlock (ScyTek), and incubated overnight with in anti-BrdU antibody in 5% normal horse serum in PBS/0.16% Triton X-100 at 4°C in blocking solution. Sections were washed in PBST, incubated with secondary antibodies in blocking solution at RT for 2 h, washed in PBST, mounted on Superfrost Plus slides (Fisher, Hampton, NH), and coverslipped with Gelvatol mounting medium.

#### Cell quantification

All tissue sections used for quantification were imaged using confocal microscopy (Meta 510, Zeiss Microscopy). ArcN and ME cells were counted. For the ME, the dorsal-ventral boundary of the cells counted was the third ventricle (3V) floor and the ventral edge of the external layer of the ME. The lateral boundaries were a 20 um medial inset off the corner of the 3V. ME dorsal and ventral boundaries remained identical to as previously described. Seven 40-μm coronal serial sections (280 μm) were counted between −1.515 and −1.875 mm from Bregma. Occasionally, a section would be lost or damaged in the collection process. If available, the next section in the mouse sample was taken and counted (seven sections were counted on average, although this could range from three to eight sections due to technical difficulties). For analysis of newborn Hu^+^ neurons, for each section analyzed, Hu^+^DAPI^+^ and Hu^+^BrdU^+^DAPI^+^ neurons within the ME were counted in the region defined above, excluding cells of the uppermost focal plane to avoid oversampling. To determine the frequency of BrdU^+^ cells expressing Hu, dual fluorescence-labeled sections were examined by confocal microscopy using a 20× objective and 1.5× digital zoom. For each marker and treatment condition, seven representative serial sections from each animal were examined. Sections were scored for double labeling by manual examination of optical slices. Cells were considered positive for a given phenotypic marker when the marker-specific labeling was unambiguously associated with a BrdU^+^ nucleus. Cells were spot-checked in all three dimensions by Z-stack using a 63× objective. Images of Hu^+^BrdU^+^DAPI^+^ labeling in feeding conditions (Figure 1B) were blinded prior to counting. Cell counts are described in the text and figure legends as mean of several samples ± s.e.m., total cell counts, and the number of samples examined to derive those total cell counts.

### Focal irradiation of ventrobasal hypothalamus

Radiation (10 Gy) was delivered using the Small Animal Radiation Research Platform (SARRP), a dedicated laboratory focal radiation device with CT capabilities (Xstrahl, Inc.). A detailed video and protocol describing this focal irradiation methodology is available online (Lee et al., 2013). In brief, C57BL6/J female mice were ordered from Jackson Mouse laboratories and housed four to a cage. At six weeks of age, mice were switched from normal to high-fat chow. Three days later, mice were divided into treatment and sham control groups and weighed. No significant differences in weight were observed between these cohorts. Mice were then gently transported to the radiation suite, taking care to minimize stress levels. One mouse from the treatment and one from the control group were then anesthetized using isoflurane gas. Heating pad set in the low setting was prepared for postoperative treatment of the mice. CT imaging was then used to identify the target, and mice were moved using robotic control to align the target with the X-ray beam. Platform rotation speed and duration were calculated, and irradiation delivered to the treatment group. Sham controls differed only in that they did not receive a direct radiation beam and CT scan, but instead remained in the anesthesia chamber. Once irradiation treatment was complete, both sham and irradiated mice were placed on a heating pad, and monitored until arousal. Mice were then returned to the animal facility and checked every day thereafter. Weights were taken twice a week.

A cohort of irradiated mice were killed within 2 h following irradiation and analyzed for γH2Ax immunostaining to confirm that double-stranded DNA breaks were confined to the median eminence, as described previously (Ford et al., 2011; Lee et al., 2012, 2013). In addition, intraperitoneal injections of BrdU (50 mg/kg) were administered beginning three days following sham or irradiation treatment. Levels of neurogenesis were determined by immunohistochemistry for BrdU and HuC/D one month following the first BrdU injection, as previously described (Lee et al., 2012).

### Longitudinal collection of weight data

Weight data from each mouse subject was collected at the time of sham or irradiation treatment. Longitudinal weight gain was normalized to weight at the time of treatment for each subject. Weight data was taken every 0.5 weeks for each mouse subject. Female mice were group housed. Male mice were all group-housed initially, but were separated if they were observed to fight. To reduce variation resulting from changes in housing, the numbers of male mice that were individually and group housed were matched at all times between the sham and irradiated cohorts. For one experiment, HFD-fed female sham or irradiated cohorts, blood samples were collected one week following treatment and a standard complete blood count panel was taken. There was no statistically significant difference in any of blood components.

### Statistical analysis

Figures are shown as mean ± standard error of the mean. Comparisons were made using Two-tailed Student's *t*-test run on Microsoft Excel 2007 or Two-Way ANOVA run on Sigmaplot 11.0 software. The *post-hoc* test, Holm-Sidak Method, was used for all pairwise multiple comparison. A *p*-value ≤ 0.05 indicated significant group difference.

### Conflict of interest statement

The authors declare that the research was conducted in the absence of any commercial or financial relationships that could be construed as a potential conflict of interest.
