# Supplementary material for: Dietary and sex-specific factors regulate hypothalamic neurogenesis in young adult mice
Source: Front Neurosci. 2014 Jun 13;8:157. doi: 10.3389/fnins.2014.00157 (PMC4056383; doi:10.3389/fnins.2014.00157)
Supplement: Supplementary file 1 [file Presentation1.PDF]

Supplementary Material

Dietary and sex-specific factors regulate hypothalamic neurogenesis in young adult mice.

Daniel A. Lee[1,2]*, Sooyeon Yoo[1]*, Thomas Pak[1]*, Juan Salvatierra[1], Esteban Velarde[3], Susan Aja[1,4], and Seth Blackshaw[1, 5-7],

[1] Solomon H. Snyder Department of Neuroscience, Johns Hopkins University School of Medicine, Baltimore, Maryland, USA.
[2] Division of Biology and Biomedical Engineering, California Institute of Technology, Pasadena, California, USA
[3] Department of Radiation Oncology and Molecular Sciences, Johns Hopkins University School of Medicine, Baltimore, Maryland, USA.
[4] Center for Metabolism and Obesity Research, Johns Hopkins University School of Medicine, Baltimore, Maryland, USA
[5] Institute for Cell Engineering, Johns Hopkins University School of Medicine, Baltimore, Maryland, USA
[6] Department of Ophthalmology, Johns Hopkins University School of Medicine, Baltimore, Maryland, USA.
[7] Center for High-Throughput Biology, Johns Hopkins University School of Medicine, Baltimore, Maryland, USA.
*These authors contributed equally to the work.

**** Correspondence:** Seth Blackshaw, Solomon H. Snyder Department of Neuroscience, Johns Hopkins University School of Medicine, Baltimore, Maryland, 21287 USA.
sblack@jhmi.edu

Supplementary Figures

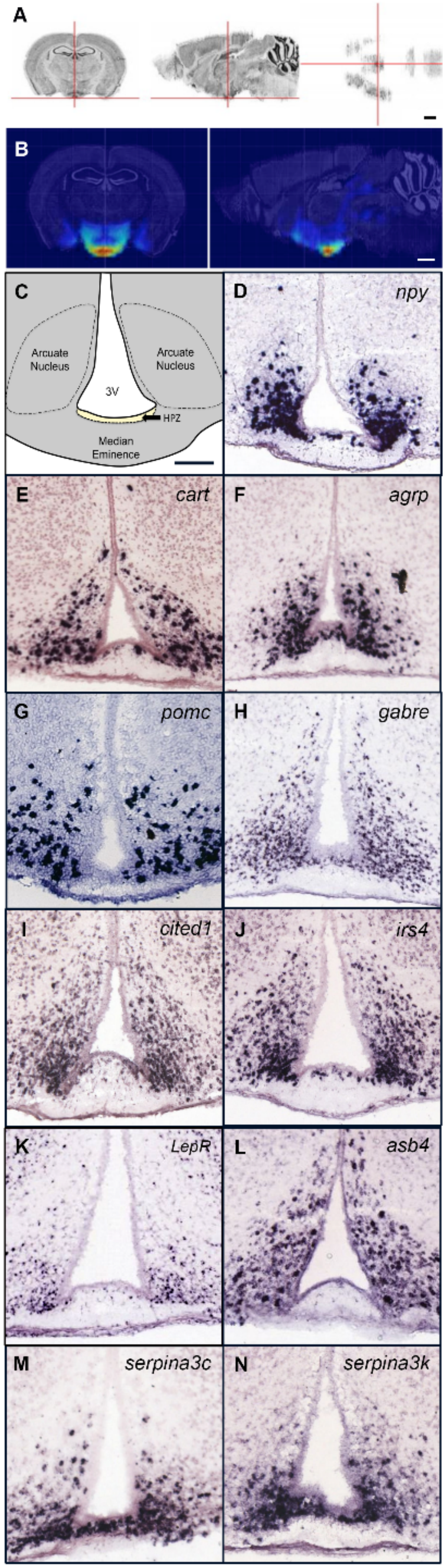

Supplemental Figure 1. Adult born mediobasal hypothalamic neuronal subtype candidates
(**A-B**) Identification of adult born hypothalamic neuronal subtypes candidate through expression profiling cluster analysis of the mediobasal hypothalamic on the *Anatomic Gene Expression Atlas* and *Allen Brain Atlas* **(C)** The mediobasal hypothalamus (MBH) is composed of the arcuate nucleus and median eminence subregions, and the hypothalamic proliferative zone (HPZ), a neurogenic region at the floor of the third ventricle (3V). (**D-N)** Candidate markers for potential adult-born MBH neuronal subtypes that may be generated in a sex- and diet-dependent manner. **(D)** neuropeptide Y mRNA (*npy*) **(E)** cocaine- and amphetamine-regulated transcript mRNA (*cart*) **(F)** agouti-related protein mRNA (*agrp*) **(G)** pro-opiomelanocortin (*pomc)* **(H)** gamma-aminobutyric acid A receptor epsilon mRNA (*gabre*) **(I)** Cbp/p300-interacting transactivator, with Glu/Asp-rich carboxy-terminal domain 1 mRNA (*cited1*) **(J)** insulin receptor substrate (*irs4*) **(K)** leptin receptor mRNA (*LepR*) **(L)** ankyrin repeat and SOCS box containing 4 mRNA (*asb4*) **(M)** serpin peptidase inhibitor, clade A, member 3c mRNA (*serpina3c*) **(N)** serpin peptidase inhibitor, clade A, member 3k mRNA (*serpina3k*) (**A-B**) Scale bar = 1mm ; (**C-N)** Scale bar = 100um. (**A-N**) Image credit: Allen Brain Atlas (http://mouse.brain-map.org/)
